# Supplementary material for: Genomic and phenotypic characterization of novel jumbo and small bacteriophages infecting Xanthomonas hortorum pv. vitians: toward a phage-based biocontrol strategy
Source: Microbiol Spectr. 2026 Jun 11;14(7):e00846-26. doi: 10.1128/spectrum.00846-26 (PMC13340338; doi:10.1128/spectrum.00846-26)
Supplement: Supplemental figures — Fig. S1 to S4. [file spectrum.00846-26-s0001.pdf]

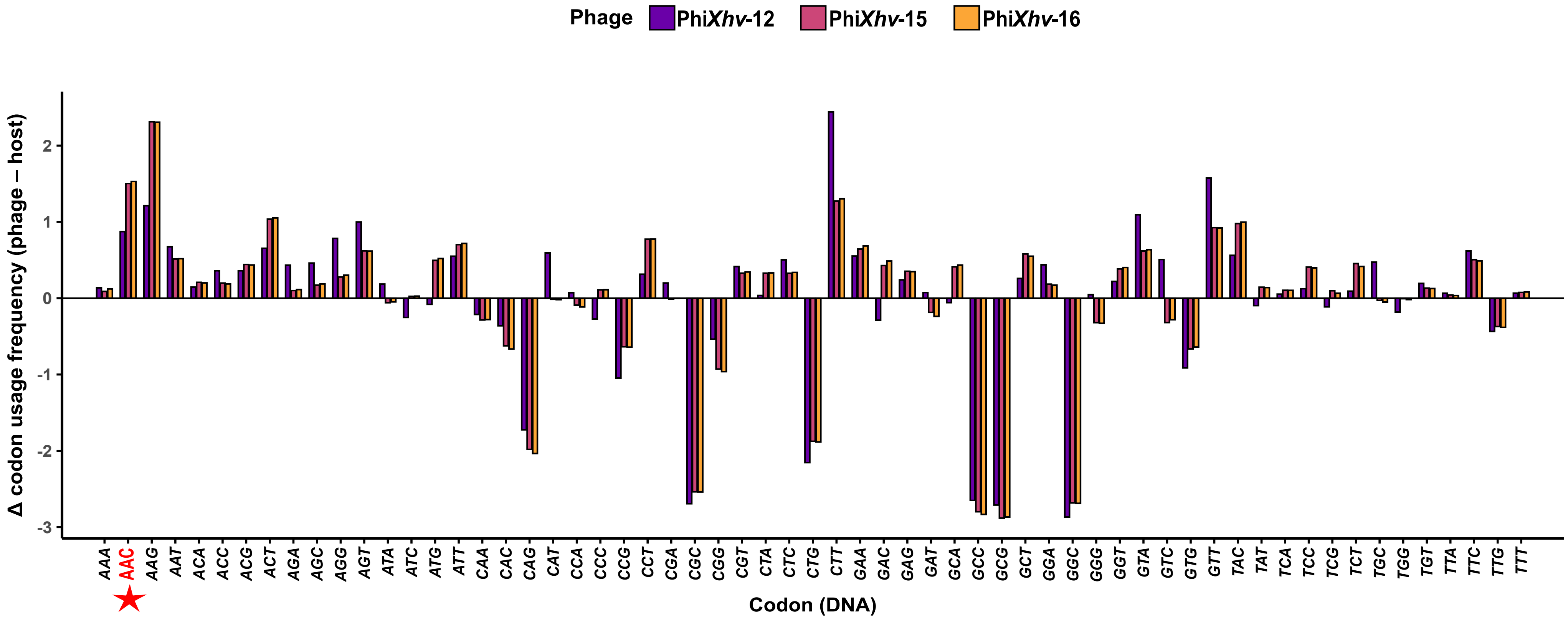

Figure S1. Codon usage bias of jumbophages relative to *X. hortorum* pv. *vitians*. The bar plot illustrates the difference in codon usage frequency (Δ frequency = phage - host) for each of the 64 DNA codon, comparing the three jumbophages (Phi*Xhv*-12, Phi*Xhv*-15, and Phi*Xhv*-16) and their host, *X. hortorum* pv. *vitians* strain CFBP8640. Positive values indicate codons more frequently used by the phage than the host, whereas negative values indicate codons less frequently used. Codons are ordered alphabetically along the x-axis. The red star highlights codon "AAC" (Asn), which corresponds to the tRNA-Asn (anticodon GTT) encoded by the jumbophages.

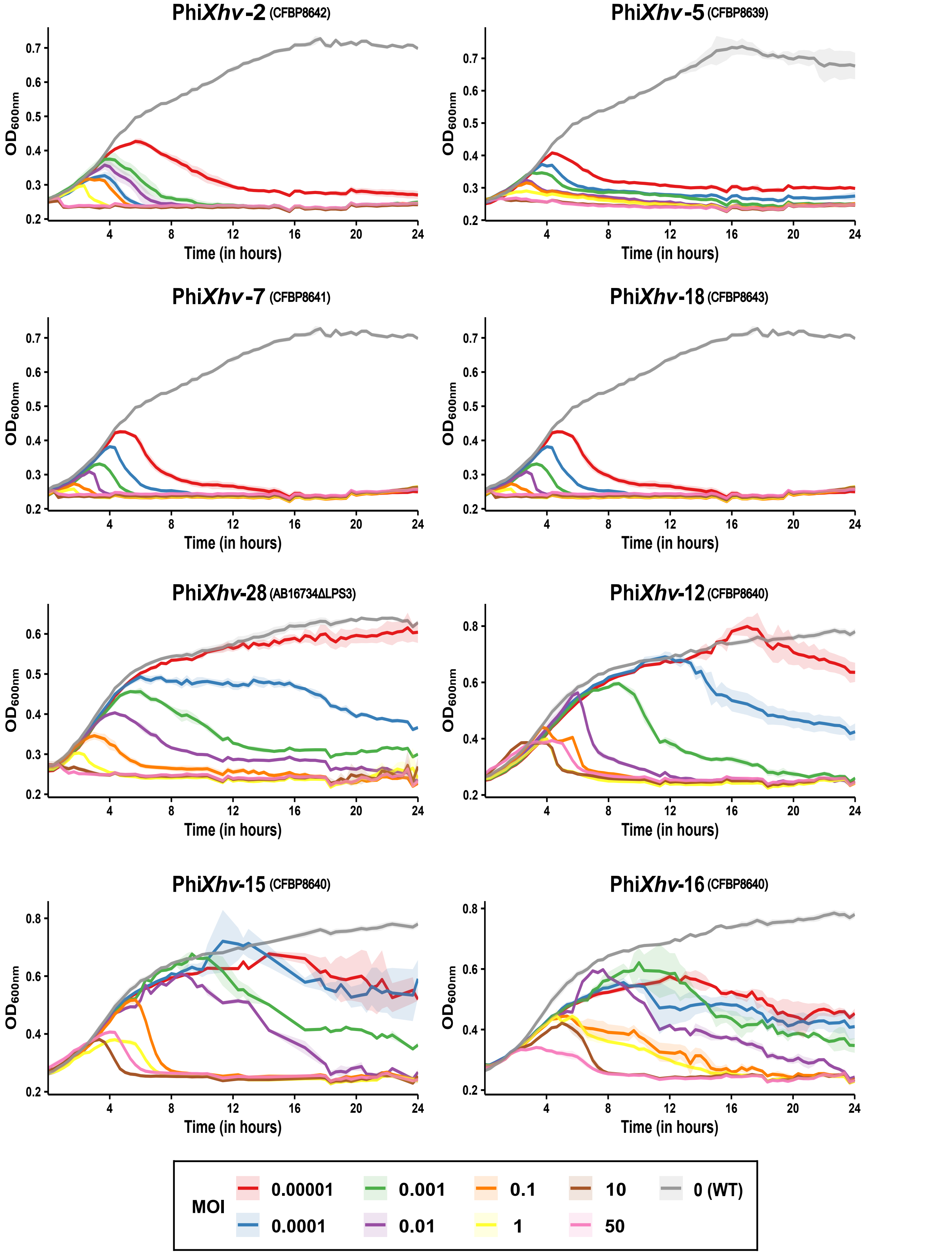

Figure S2. Growth kinetics of *X. hortorum* pv. *vitians* after infection by phages at different multiplicities of infection (MOIs). Each panel displays the mean bacterial growth curve measured by optical density at 600nm (OD_{600nm}) over 24 h at 28°C for a specific phage (Phi*Xhv*-2, Phi*Xhv*-5, Phi*Xhv*-7, Phi*Xhv*-12, Phi*Xhv*-15, Phi*Xhv*-16, Phi*Xhv*-18, or Phi*Xhv*-28). Phages were added at MOIs ranging from 0.00001 to 50, as indicated by the color-coded legend. Phage-free controls (0 MOI, Wild Type -WT) are shown in grey. Shaded areas represent the standard deviation from four technical replicates per condition. Strain names used for phage propagation are indicated in brackets.

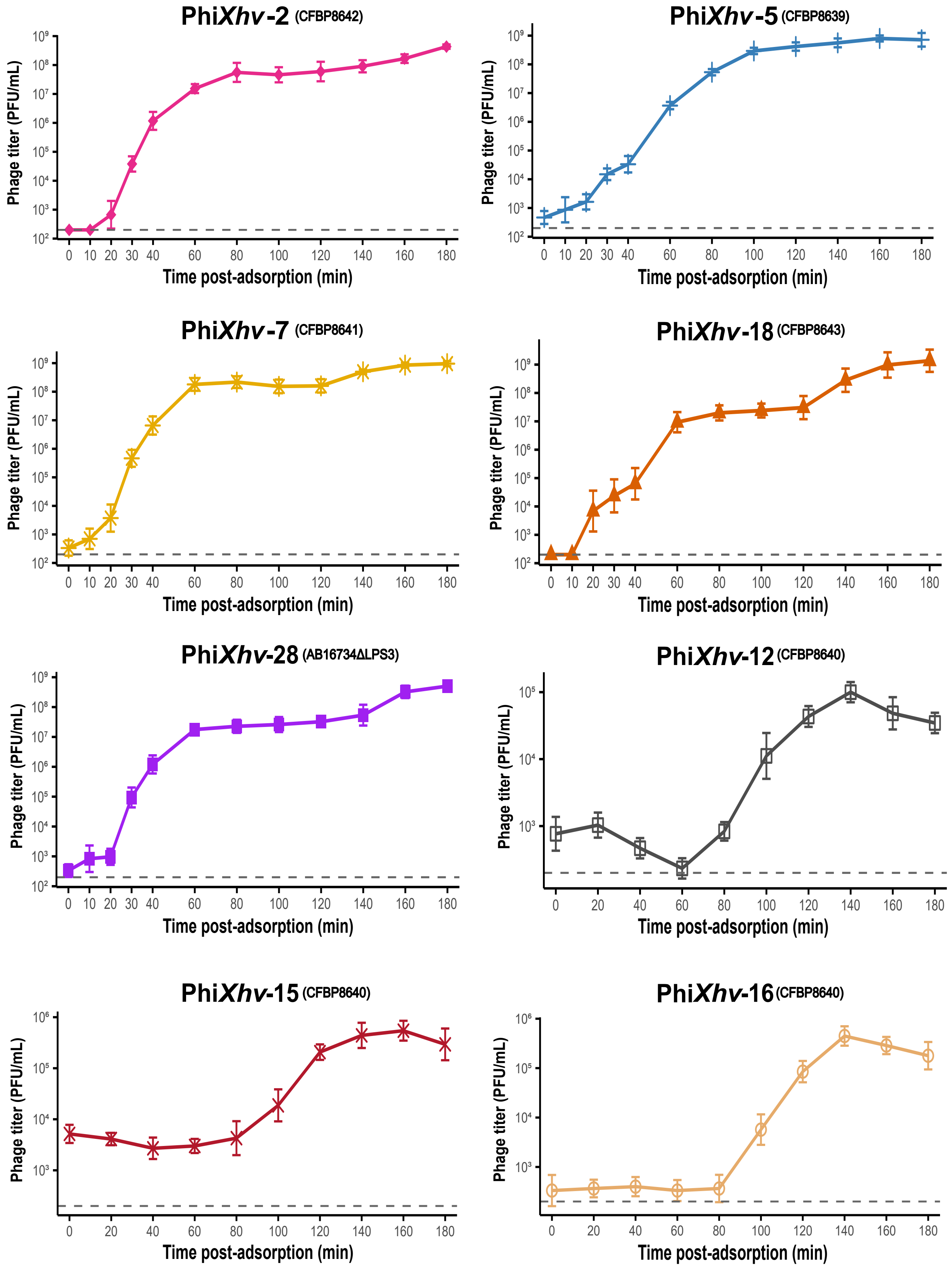

Figure S3. One-step growth curves of phages infecting *X. hortorum* pv. *vitians*. Each panel shows the phage titer (PFU/mL) measured over time following infection of exponentially growing *X. hortorum* pv. *vitians* cultures with individual phages: Phi*Xhv*-2, Phi*Xhv*-5, Phi*Xhv*-7, Phi*Xhv*-12, Phi*Xhv*-15, Phi*Xhv*-16, Phi*Xhv*-18, and Phi*Xhv*-28. Bacterial cells were infected at a MOI of 0.1 or 0.001 (for jumbophages) and incubated at 28°C. Time post-adsorption refers to time elapsed after the adsorption phase and two successive centrifugation steps (20 min), used to remove non-adsorbed phage. Samples were collected at regular intervals, and the number of infection particles released was determined by spot assay. Dashed lines indicate the assay detection limit. Data points represent mean ± standard deviation from two independent biological replicates, each with three technical replicates per time point. Strain names used for phage propagation are indicated in brackets.

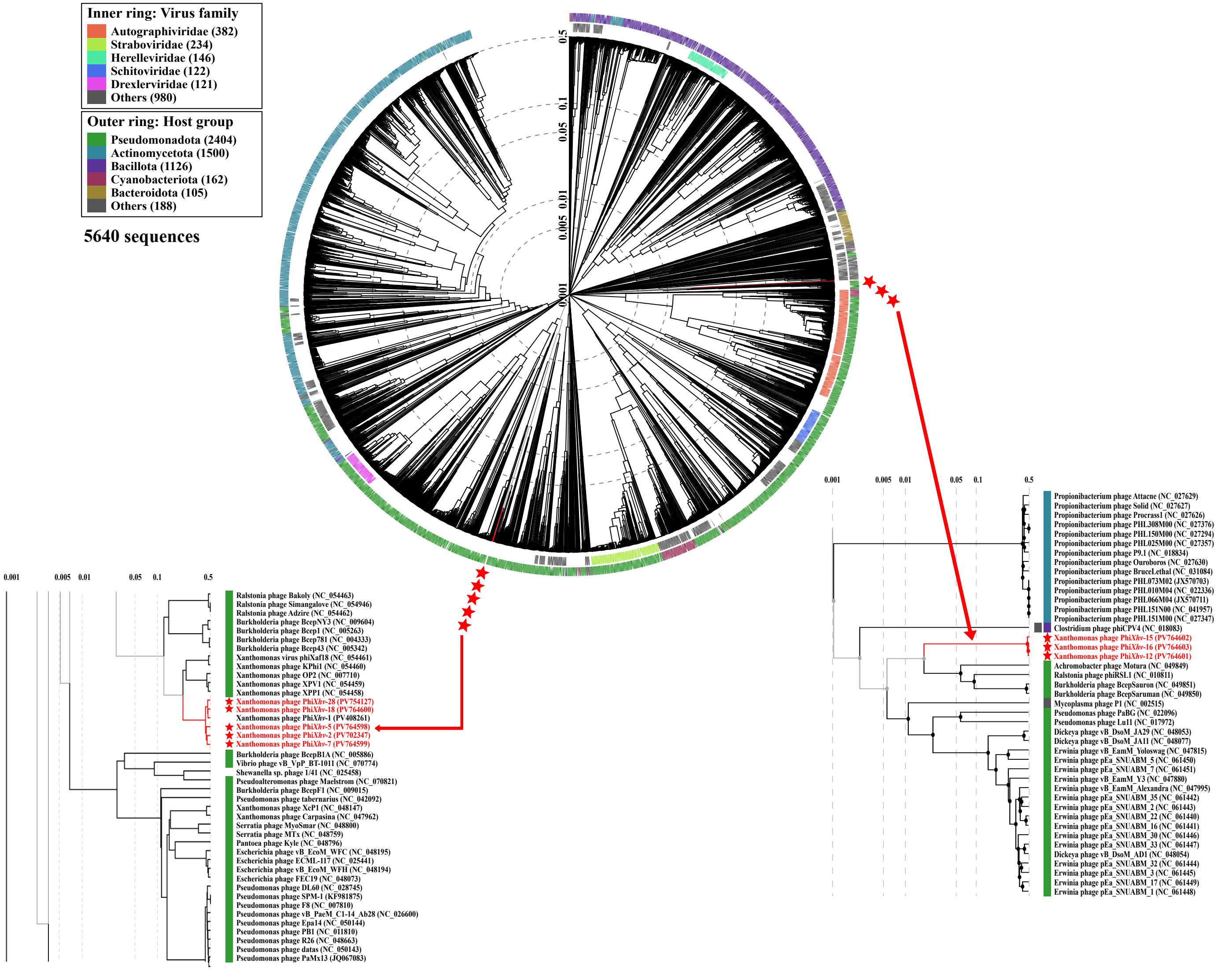

Figure S4. Proteomic analysis of *X. hortorum* pv. *vitians* bacteriophages within a global context of phage diversity. This proteomic dendrogram depicts the proteomic relationships of eight newly discovered *X. hortorum* pv. *vitians* phage genomes (indicated by red stars) relative to 5,640 reference phage genomes. The proteomic dendrogram was generated with ViPTree and edited using Inkscape v1.4. The inner ring indicates virus family assignments, while the outer ring shows host group classifications of these phages. Two zoomed-in regions highlight the relationships of the *X. hortorum* pv. *vitians* phages relative to closely related viruses. Branch lengths represent genome-wide proteomic distances. Phages are labeled with their respective GenBank accession numbers.
