# Supplementary material for: Genomic and phenotypic characterization of novel jumbo and small bacteriophages infecting Xanthomonas hortorum pv. vitians: toward a phage-based biocontrol strategy
Source: Microbiol Spectr. 2026 Jun 11;14(7):e00846-26. doi: 10.1128/spectrum.00846-26 (PMC13340338; doi:10.1128/spectrum.00846-26)
Supplement: Supplemental material — Additional experimental details. [file spectrum.00846-26-s0002.docx]

**Genomic and phenotypic characterization of novel jumbo and small bacteriophages infecting *Xanthomonas hortorum* pv. *vitians*: Toward a phage-based biocontrol strategy**

**Anaelle Baud^1,*^, Inès Rougis^1^, Nicolas Taveau^2^, Denis Costechareyre^2^, Marie Graindorge Beaume^2^, Franck Bertolla^1,*^**

**Author affiliations**:

^1^ Université Lyon 1, CNRS, INRAE, LEM, UMR 5557, UMR 1418 Villeurbanne, France.

^2^ GREENPHAGE, 34830 Clapiers, France.

***Correspondence:** Franck Bertolla, franck.bertolla@univ-lyon1.fr; Anaelle Baud, anaelle.baud@univ-lyon1.fr

**Supplemental material**

**Strains and growth conditions**

*Xanthomonas* strains used in this study are listed in **Table S1**. Strains were routinely streaked onto tryptic soy agar (TSA) plates and incubated at 28°C for 48-72 h. Single colonies were used to inoculate tryptic soy broth (TSB) and cultured overnight at 28°C with shaking at 160 rpm. Strains of *X. hortorum* pv. *vitians* used for phage isolation were listed in **Table S1**. Semi-solid agar overlays consisting of TSB with 0.6% agar (or 0.4% for jumbophages), supplemented with 1 mM CaCl_2_ to promote phage adsorption, were used for plaque assays. For phage infection experiments, overnight bacterial cultures were diluted in TSB supplemented with 10 mM CaCl_2_ to an optical density at 600 nm (OD_600_) of 0.15 (7.5 × 10^7^ CFU.mL^-1^) and incubated at 28°C with shaking at 160 rpm for 1 hour to reach the early logarithmic phase prior to phage addition. All strains were kept at −80 °C in 30% (v/v) glycerol vials for long-term storage.

**Phage isolation, purification, and propagation**

A total of 42 samples were collected between November 2022 and December 2024 from various wastewater treatment plants located in the Occitanie region (France) for the isolation of *X*. *hortorum* pv. *vitians* phages. Effluent samples were centrifuged at 5,000 x *g* for 10 min at room temperature, and the resulting supernatants were filtered through a 0.2 μm filter to remove debris. For enrichment, 3 mL of filtered effluent were mixed with 7 mL of early logarithmic-phase cultures of different *X. hortorum* pv*. vitians* strains. For each enrichment series, a host-only control was included, in which the bacterial strain was incubated without any environmental sample under identical conditions. After overnight incubation (160 rpm, 28 °C), the cultures were reprocessed by centrifugation and filtration as described above. The presence of phages was evaluated using the double-layer agar technique, where the appearance of individual plaques indicated successful phage isolation. Briefly, 100 µL of enrichment supernatant were mixed with 500 µL of an overnight culture of *X. hortorum* pv*. vitians* and 4 mL of molten soft TSB overlay supplemented with 1 mM CaCl_2_, then poured onto TSA plate. After 18 h at 28 °C, individual plaques were picked using filter tips and transferred into 2.5 mL of SM buffer (50 mM Tris-HCl [pH 7.5], 8 mM MgSO_4_.7H_2_O, 100 mM NaCl). The mixture was gently agitated at 4°C for 1 h and then filtered through a 0.2 µm filter. This plaque-purification step was repeated twice to obtain clonal phage isolates. Phage stocks were stored at 4°C in SM buffer. Plaque morphology (i.e., size, clarity, turbidity, edge definition) was evaluated using the single agar layer method. Unless otherwise specified, all phage suspensions used in the following experiment were filtered through 0.2 µm pore-size filters prior to use.

Phages were propagated using the double-layer agar technique. Briefly, 100 µL of purified phage suspension (~10^8^ PFU/mL) and 100 µL of an overnight culture of the isolation strain were mixed with 4 mL of molten soft TSA overlay, then poured onto the TSA plates and incubated overnight at 28°C. Plates with confluent lysis were flooded with 7 mL of SM buffer, and the resuspended soft top agar was collected and incubated at 28°C with shaking at 150 rpm for 2 h to release phage particles. The lysate was centrifuged at 4,000 x *g* for 7 min, and the supernatant was filtered twice before storage at 4°C. Phage titers (in PFU/mL) were determined using the spot assay. Briefly, 10 µL of serial tenfold dilutions were spotted onto soft TSA overlays seeded with the host strain.

For large-scale production, each phage was amplified in liquid culture. An early logarithmic-phase culture (OD_600_ ~ 0.2, equivalent to 1.10^8^ CFU.mL^-1^) of the production strain was mixed with phage at a multiplicity of infection (MOI) of 0.1 in TSB supplemented with 10 mM CaCl_2_. Cultures were incubated for up to 16 h at 28°C with shaking at 160 rpm. Following lysis, cultures were centrifuged at 5,000 x *g* for 10 min at 4°C, and the clear supernatants were filtered. Final titers were determined by spot assay on double-layer agar.

**Phage morphological characterization by transmission electronic microscopy (TEM)**.

Phage particles were sedimented by centrifugation (21,800 × *g*, 90 min, 4 °C), and the pellet was further washed twice with 0.1 M acetate ammonium buffer by repeating the centrifugation step. Subsequently, phage suspensions were dried on a 300-mesh formvar-coated grid (Delta Microscopies) for 2 min at room temperature, and excess solution was removed using filter paper. Grids were negatively stained with 2% uranyl acetate. Grids were washed with two 10µL-droplets of uranyl acetate 2 % and then stained by incubation with one 10µL-droplet of uranyl acetate 2 % for 30 s. Excess of uranyl acetate was drained on a blotting paper and grids were dried for 10 min before image acquisition. The grids were imaged via TEM using a JEOL 1400 Flash microscope operated at 120 kV. Virion dimensions (capsid diameter, tail length/width) were measured from at least ten individual virions micrographs using ImageJ software (v.154d) (1). Results are reported as a mean value ± standard error.

**Phage whole-genome sequencing and analysis**

Phage DNA was extracted and purified as described previously by Gendre J (2) without addition of SDS and proteinase K. Libraries were prepared using the Illumina TruSeq PCR-Free and Nextera XT DNA Library Prep Kits, and sequencing was performed on an Illumina Miseq platform or NovaSeq 6000 S4 platforms (paired-end, 150 bp), depending on the phage sample. Reads quality was assessed using FastQC v0.12.1 (3) followed by a cleaning using PRINSEQ 0.20.4 (4). Reads were assembled using SPAdes 4.0 (5). Contigs sequences were queried against the NCBI nucleotide (nt) database using BLASTn (6) to confirm viral origin and exclude contaminant sequences. Assembly quality was evaluated by read mapping using Bowtie2 v2.5.4 (7), followed by processing with Samtools v1.20 (8). Structural and functional annotation was carried out using the rTOOLS pipeline (RIME Bioinformatics). Transfer RNAs were identified using tRNA scan-SE v2.07 (9). Phage lifestyle was predicted using PhageAI (10). Comparative genomic analysis among phage isolates was performed using CLINKER (11). To explore functional genomic divergence within the two proposed phage genera, predicted protein sequences were compared using an all-versus-all BLASTp approach. Sequences were clustered into protein families using SiLiX based on pairwise similarities (12). Proteins were assigned to the same family if their high-scoring segment pairs (HSPs) covered at least 80% of the protein length and shared ≥ 35% sequence identity, as previously described (13). Partial sequences were included if they were ≥ 100 amino acids in length or represented at least 50% of the full-length protein. Presence/absence matrices were generated using custom scripts. Phylogenomic relationships were inferred with ViPTree (14), which constructs a proteomic tree based on genome-wide tBLASTx similarities against reference viral genomes. Intergenomic similarities among complete phage genomes were calculated using VIRIDIC with Blastn default settings (15).

**Host range determination**

The plaquing host range of the phages was assessed by spot assays on 41 *Xanthomonas* strains, including 34 *X*. *hortorum* pv. *vitians*, five strains from other pathovars and two from other species (**Table S1**). Briefly, for each strain, 900 µL of log-phase bacterial culture were mixed with 12.5 mL of molten soft TSB agar and poured into square Petri dishes. After solidification, 5 µl drops of 10-fold serially diluted phage suspensions in sterile distilled water (dH_2_O) were spotted onto the overlay. Plates were incubated overnight at 28°C. A strain was considered susceptible to a phage, if clear countable plaques were observed. Lysis from without (halo without plaques) or absence of lysis indicated resistance. Efficiency of plating (EOP) was calculated as the ratio of the phage titer (in PFU/mL) on the test strain to the titer on the production host. Based on EOP values, strains were classified as highly sensitive (EOP > 0.5), sensitive (10 ^-4^ < EOP < 0.5) or resistant (EOP < 10 ^-4^). This classification was adapted from previously established EOP categories, using a lower resistance threshold (EOP < 10^-4^ instead of 10^-3^) (16, 17). As a negative control, each bacterial overlay was also spotted with 5 µL of sterile dH_2_O. All experiments were done in duplicate, with two technical replicates per phage dilution. The relationship between phage genome size (categorized as small or large) and host range breadth was analyzed using a chi-squared test of independence. To evaluate whether genetic diversity among *X*. *hortorum* pv. *vitians* strains influenced phage susceptibility, differences in overall susceptibility scores across MLSA-defined groups (A, B, and C) were tested using the Kruskal-Wallis rank-sum test. Group-specific biases in susceptibility at the individual phage level were assessed using Fisher’s exact tests with Monte Carlo simulation (10,000 replicates, to approximate p-values), followed by Bonferroni correction for multiple testing.

**Bacterial growth inhibition assay**.

The ability of phages to inhibit the growth of their production strain was assessed at various MOIs by monitoring OD_600_ using a Bioscreen C MBR BACTERIO (Thermo Fisher Scientific, Illkirch-Graffenstaden, France). Briefly, 170 µL of an early exponential-phase host culture (OD_600_ ~ 0.2, equivalent to 10^8^ CFU.mL^-1^) were inoculated with phage suspensions at MOIs of 0.00001, 0.0001, 0.001, 0.01, 0.1, 1, 10, and 50 in 100-well Honeycomb plates. Plates were incubated for 30 min at room temperature to allow phage adsorption. Wells inoculated with TSB only and bacterial strain without phages served as negative and bacterial growth controls, respectively. OD_600_ was recorded every 20 min for 24 h at 28°C under continuous orbital shaking (normal speed, medium amplitude). Four technical replicates per treatment were performed.

**Adsorption assays**

Phage adsorption kinetics were determined by quantifying non-adsorbed phages over time. Early-log-phase culture of production strains (OD_600_ ≈ 0.2, 10^8^ CFU.mL^-1^) in 30 mL TSB supplemented with 10 mM CaCl_2_ were mixed with phages at a MOI of 10^-2^ (10^6^ PFU/mL). The co-cultures were incubated at 28°C. For jumbophages, gentle agitation (50 rpm) was applied. This condition was selected based on preliminary observations showing that static incubation significantly reduced jumbo phage adsorption efficiency. Aliquots (1 mL) were collected every 10 min for 1 h or every 30 min for 2 h in the case of jumbophages. Samples were filtered through 0.2 µm pore-size filters to collect non-absorbed (free) phages. Five-microliters drops of serial 10-fold dilutions were spotted onto soft TSB agar overlays containing the production strain to quantify free phages, after overnight incubation at 28°C. Two independent experiments were performed, each consisting of three technical replicates for phage titration. At each time point, the percentage of free phages was calculated as the titer at that time relative to initial titer. Adsorption rate constant (*k*) was calculated as described previously (18).

**One-step growth curves**

One-step growth curves were determined to assess the infectivity and replication dynamics of the phages. Each phage was added at a MOI of 10^-1^ or 10^-3^ (for jumbophages) to an early log-phase culture (OD_600_ ~ 0.2, ~10^8^ CFU.mL^-1^) of the production strain. Co-cultures were incubated at 28°C (with gentle agitation at 50 rpm for jumbophages) for the previously determined adsorption time. Aliquots were collected before and after the adsorption step. Non-adsorbed phages were removed by centrifugation (5,000 x *g*, 21°C, 10 min), the supernatant was discarded, and the pellets were resuspended in 35 mL of TSB supplemented with 10 mM CaCl2. This wash step was performed twice to remove non-adsorbed phages. The resuspended co-cultures were incubated at 28°C with shaking at 160 rpm. Aliquots were collected over a period of 3 h. Samples were filtered to isolate free phage particles. Phage titers at each time point were determined using the spot assay as described above. The burst size was calculated as the ratio of the number of phages released after the rise period (corrected by subtracting the phage titer at t_0_) to the number of adsorbed phages. The number of adsorbed phages was determined by subtracting the titer of non-adsorbed phages remaining after the adsorption step from the initial phage input. Two independent experiments were performed, each with three technical replicates per time point.

**Effect of temperature, pH, and ultraviolet irradiation on phages stability**

To assess phage stability, suspensions were subjected to various abiotic conditions including temperature, pH, and UV-B irradiation. For thermal stability, each phage suspension (10^6^ PFU/mL in TSB) was incubated in the dark for 1 h at 4°C, 28°C, 37°C, 55°C, 65°C and 75°C in a thermal cycler (Biometra TOne, Analytik Jena, Jena, Allemagne). pH stability was evaluated by mixing 100 µL of phage suspension (10^7^ PFU/mL) with 900 µL of SM buffer adjusted to pH values of 2, 4, 6, 7.5, 8, 10, or 12 using HCl or KOH. Samples were incubated at room temperature in the dark for 1 h. For UV sensitivity assays, 150 µL of the phage suspension (10^6^ PFU/mL) were poured into sterile empty 60 mm Petri dishes and exposed to UV-B light emitted by a Bio-Link Crosslinker BLX-E312 (Vilber Lourmat, Collégien, France). Dishes were left open and placed at a 16 cm distance from the light source (5 x 8W, 120 mW/cm2, emission peak at 312 nm) during 0, 2, 5, 10, 15 or 30 min. Phage viability following each treatment was assessed by plaque counting using the double-layer agar method. The phage survival rate (%) was calculated as the ratio of the phage titer after treatment to the initial titer, measured under optimal storage conditions (4°C, pH 7.5, in the dark). Each abiotic condition was tested in two independent experiments, each with three technical replicas.

**Data analysis and visualization.**

All figures were generated using R (v4.3.3; R Core Team) within RStudio (version 2023.12.1.402 “Ocean Storm” Release; RStudio Team), unless otherwise specified. Visualizations were primarily created using the ggplot2 package (v3.5.1), with additional packages including readxl (v1.4.3) for data import, dplyr (v1.1.4) for data manipulation, and patchwork (v1.3.0) for figure assembly. Specific visualizations, such as heatmaps, employed ComplexHeatmap (v2.18.0) and circlize (version 0.4.16). Other packages used for figure-specific processing include scales (v1.3.0), tidyr (v1.3.1), purr (v1.0.2), cowplot (v1.1.3), and viridis (v0.6.5). When specified, figures were post-processed for layout optimization and labeling using Inkscape (v1.4). Figures were exported in high resolution (1200 dpi) in SVG, PNG, and/or PDF formats for publication purposes.

**REFERENCES**

1. Schneider CA, Rasband WS, Eliceiri KW. 2012. NIH Image to ImageJ: 25 years of image analysis. Nat Methods 9:671–675.

2. Gendre J, Ansaldi M, Olivenza DR, Denis Y, Casadesús J, Ginet N. 2022. Genetic Mining of Newly Isolated Salmophages for Phage Therapy. Int J Mol Sci 23:8917.

3. Wingett SW, Andrews S. 2018. FastQ Screen: A tool for multi-genome mapping and quality control. F1000Res 7:1338.

4. Schmieder R, Edwards R. 2011. Quality control and preprocessing of metagenomic datasets. Bioinformatics 27:863–864.

5. Nurk S, Bankevich A, Antipov D, Gurevich AA, Korobeynikov A, Lapidus A, Prjibelski AD, Pyshkin A, Sirotkin A, Sirotkin Y, Stepanauskas R, Clingenpeel SR, Woyke T, McLean JS, Lasken R, Tesler G, Alekseyev MA, Pevzner PA. 2013. Assembling single-cell genomes and mini-metagenomes from chimeric MDA products. J Comput Biol 20:714–737.

6. Camacho C, Coulouris G, Avagyan V, Ma N, Papadopoulos J, Bealer K, Madden TL. 2009. BLAST+: architecture and applications. BMC Bioinformatics 10:421.

7. Langmead B, Salzberg SL. 2012. Fast gapped-read alignment with Bowtie 2. Nat Methods 9:357–359.

8. Li H, Handsaker B, Wysoker A, Fennell T, Ruan J, Homer N, Marth G, Abecasis G, Durbin R, 1000 Genome Project Data Processing Subgroup. 2009. The Sequence Alignment/Map format and SAMtools. Bioinformatics 25:2078–2079.

9. Chan PP, Lin BY, Mak AJ, Lowe TM. 2021. tRNAscan-SE 2.0: improved detection and functional classification of transfer RNA genes. Nucleic Acids Res 49:9077–9096.

10. Tynecki P, Guziński A, Kazimierczak J, Jadczuk M, Dastych J, Onisko A. 2020. PhageAI - Bacteriophage Life Cycle Recognition with Machine Learning and Natural Language Processing. bioRxiv https://doi.org/10.1101/2020.07.11.198606.

11. Gilchrist CLM, Chooi Y-H. 2021. clinker & clustermap.js: automatic generation of gene cluster comparison figures. Bioinformatics 37:2473–2475.

12. Miele V, Penel S, Duret L. 2011. Ultra-fast sequence clustering from similarity networks with SiLiX. BMC Bioinformatics 12:116.

13. Penel S, Arigon A-M, Dufayard J-F, Sertier A-S, Daubin V, Duret L, Gouy M, Perrière G. 2009. Databases of homologous gene families for comparative genomics. BMC Bioinformatics 10:S3.

14. Nishimura Y, Yoshida T, Kuronishi M, Uehara H, Ogata H, Goto S. 2017. ViPTree: the viral proteomic tree server. Bioinformatics 33:2379–2380.

15. Moraru C, Varsani A, Kropinski AM. 2020. VIRIDIC-A Novel Tool to Calculate the Intergenomic Similarities of Prokaryote-Infecting Viruses. Viruses 12:1268.

16. Wang R, You X, Liu X, Fei B, Li Y, Wang D, Zhu R, Li Y. 2024. Characterization of phage HZY2308 against Acinetobacter baumannii and identification of phage-resistant bacteria. Virol J 21:283.

17. Mirzaei MK, Nilsson AS. 2015. Isolation of Phages for Phage Therapy: A Comparison of Spot Tests and Efficiency of Plating Analyses for Determination of Host Range and Efficacy. PLOS ONE 10:e0118557.

18. Bacteriophages : Adams, Mark Hancock, 1912-1956 : Free Download, Borrow, and Streaming : Internet Archive. https://archive.org/details/bacteriophages00adam/page/n5/mode/2up. Retrieved 2 August 2025.
