# Supplementary material for: Genomic and phenotypic characterization of novel jumbo and small bacteriophages infecting Xanthomonas hortorum pv. vitians: toward a phage-based biocontrol strategy
Source: Microbiol Spectr. 2026 Jun 11;14(7):e00846-26. doi: 10.1128/spectrum.00846-26 (PMC13340338; doi:10.1128/spectrum.00846-26)
Supplement: Reviewer comments [file reviewer-comments.pdf]

AMERICAN SOCIETY FOR MICROBIOLOGY | Microbiology Spectrum

Genomic and phenotypic characterization of novel jumbo and small bacteriophages infecting Xanthomonas hortorum pv. vitians: Toward a phage-based biocontrol strategy

Anaelle Baud, Inés Rougis, Nicolas Taveau, Denis Costechareyre, Marie Graindorge Beaume, and Frank Bertolla

Corresponding Author(s): Frank Bertolla, Universite de Lyon

Review Timeline:

Submission Date:	March 12, 2026
Editorial Decision:	April 21, 2026
Revision Received:	May 6, 2026
Accepted:	May 8, 2026

Editor: Olaya Rendueles

Reviewer(s): Disclosure of reviewer identity is with reference to reviewer comments included in decision letter(s). The following individuals involved in review of your submission have agreed to reveal their identity: Alexander N Ignatov (Reviewer #2)

Transaction Report:

(Note: With the exception of the correction of typographical or spelling errors that could be a source of ambiguity, letters and reports are not edited. The original formatting of letters and referee reports may not be reflected in this compilation.)

DOI: https://doi.org/10.1128/spectrum.00846-26

Re: Spectrum00846-26 (Genomic and phenotypic characterization of novel jumbo and small bacteriophages infecting Xanthomonas hortorum pv. vitians: Toward a phage-based biocontrol strategy)

Dear Dr. Frank Bertolla:

Thank you for the privilege of reviewing your work. Your manuscript is very close to publication. Il y minor changes are required. Below you will instructions from the Spectrum editorial office, and the reviewer comments.

Please return the manuscript within 60 days; if you cannot complete the modification within this time period, please contact me. If you do not wish to modify the manuscript and prefer to submit it to another journal, notify me immediately so that the manuscript may be formally withdrawn from consideration by Spectrum.

Revision Guidelines
To submit your modified manuscript, log into the submission site at https://spectrum.msubmit.net/cgi-bin/main.plex. Go to Author Tasks and click the appropriate manuscript title to begin. The information you entered when you first submitted the paper will be displayed; update this as necessary. Note the following requirements:

• Upload point-by-point responses to the issues raised by the reviewers in a file named "Response to Reviewers," NOT in your cover letter.
• Upload a compare copy of the manuscript (without figures) as a "Marked-Up Manuscript" file.
• Upload a clean .DOC/.DOCX version of the revised manuscript and remove the previous version.
• Each figure must be uploaded as a separate, editable, high-resolution file (TIFF or EPS preferred), and any multipanel figures must be assembled into one file.
• Any supplemental material intended for posting by ASM should be uploaded with their legends separate from the main manuscript. You can combine all supplemental material into one file (preferred) or split it into a maximum of 10 files with all associated legends included.

For complete guidelines on revision requirements, see our Submission and Review Process webpage. Submission of a paper that does not conform to guidelines may delay acceptance of your manuscript.

Data availability: ASM policy requires that data be available to the public upon online posting of the article, so please verify all links to sequence records, if present, and make sure that each number retrieves the full record of the data. If a new accession number is not linked or a link is broken, provide Spectrum production staff with the correct URL for the record. If the accession numbers for new data are not publicly accessible before the expected online posting of the article, publication may be delayed; please contact production staff (Spectrum@asmusa.org) immediately with the expected release date.

Publication Fees: For information on publication fees and which article types are subject to charges, visit our website. If your manuscript is accepted for publication and any fees apply, you will be contacted separately about payment during the production process; please follow the instructions in that e-mail. Arrangements for payment must be made before your article is published.

ASM Membership: Corresponding authors may join or renew ASM membership to obtain discounts on publication fees. Need to upgrade your membership level? Please contact Customer Service at Service@asmusa.org.

The ASM Journals program strives for constant improvement in our submission and publication process. Please tell us how we can improve your experience by taking this quick Author Survey.

Thank you for submitting your paper to Spectrum.

Sincerely,
Olaya Rendueles
Editor
Microbiology Spectrum

Reviewer #1 (Comments for the Author):

After the first round of review, Baud et al manuscript seems very clear to me and appropriate in terms of content. I have only few recommendations to improve a bit this nice reported work.

• Given that temperate phages are also able to trigger lytic cycles, I recommend to replace "lytic phages" and even "strictly lytic phages" by "virulent phages" throughout the manuscript, as L. 54, 55, 78, 140,389, 455, 457, 587, 595, 648.

• L.58: I recommend to replace "compare to the smaller genome phages by "compare to the five smaller genome phages"
• L.111: I would reformulate "Indeed, phages present unique..." by "Indeed, phages, and specifically virulent phages, present unique..."
• L.145: replace 3 by three
• L.160 and 162: is the difference of CaCl2 concentration ok?
• L.225: replace blastn by Blastn
• L.228: replace lytic host range by plaquing host range
• L.308: replace structures by morphotypes
• L.339: I would write "...average genome depth coverage..."
• L.363: replace 7 by seven. Same L.621
• L.443 and 444: I recommend not using the terms "phylogenetic relationships" and "phylogenetic tree" but the terms whole proteomic relationships and proteomic dendrogram, given VIPTree is based on whole proteome similarity. Same comment concerning FigS4 legend.
• L.485: I would write "... protein families..."
• L.487: I recommend qualifying a bit the statement, by writing "..., potentially pointing..."
• L.556: I would write "jumbo myophages" instead of "myovirus jumbophages", as you do L.560. In this case, the word myoviruses (or myophages) does not need to be in italics.
• L.597: For is in bold, remove this.
• L.639: I would write "...eight novel virulent phages..." and "...the first three..."
• L.644: I would write "...the five smaller genome phages..."

Reviewer #2 (Comments for the Author):

The manuscript describes eight novel lytic tailed phages isolated from wastewater samples, including three jumbophages, which are the first known to infect all X. hortorum pv. vitians. These jumbophages define a new viral genus and exhibit distinct biological characteristics, including broader host ranges, but lower burst sizes and slower lytic cycles compared to the smaller-genome phages.
The newly isolated phages exhibited lytic activity against nearly all X. hortorum pv.vitians strains tested and were highly specific to the pathovar. Genomic analyses confirmed lytic profiles, and absence of virulence or resistance genes, supporting their biosafety for agricultural use. The phages demonstrated high stability across a wide range of pH and temperature conditions, remaining highly sensitive to UV exposure. These findings provide a valuable foundation for developing phage-based biocontrol strategies against bacterial leaf spot of lettuce.
The manuscript has been improved according to Reviewers 1 and 2 comments. There is only one advice for this text:

Line 102 "Their potential was recognized as early as 1924, when Hemstreet and Mallmann used a filtrate from decomposed cabbage containing phages to treat Xanthomonas campestris pv. campestris (12).

It is a general mistake, met in several reviewed manuscripts: In reference 12 Mallmann W.L, Hemstreet C. 1924. Isolation of an inhibitory substance from plants., p. 599-602 698 pp.. Journal of Agricultural Research, the Authors have described bacteriophages of soft rot pathogens, not of the black rot agent Xanthomonas campestris.

Citation from Mallmann W.L, Hemstreet C. (1924): "Pure cultures were obtained of Bacillus coli, B. carotovorus, B. melonis, and an organism closely resembling B. carotovorus but apparently belonging to the fluorescent group, which is responsible for a soft rot of cabbage. Varying amounts (up to 2 c.c.) of the cabbage filtrate rot were added after passing through a Berkefeld filter to each of these cultures, and subsequently to those of B. spieckermani and the 'potato rot bacillus'. The inhibitory effects of the filtrate on the cabbage rot organism were immediately apparent, growth being retarded for 48 hours. After repeated transfers the inhibitory substance also adapted itself to B. spieckermani and B. carotovorus but produced no effect on the potato rot organism.". Please, correct this sentence.

Response to Reviewers

Response to Reviewer #1

Reviewer #1 (Comments for the Author):

After the first round of review, Baud et al manuscript seems very clear to me and appropriate in terms of content. I have only few recommendations to improve a bit this nice reported work.
We thank the reviewer for the careful evaluation of our manuscript and for the constructive suggestions, which have helped improve the clarity and precision of the text.

• Given that temperate phages are also able to trigger lytic cycles, I recommend to replace "lytic phages" and even "strictly lytic phages" by "virulent phages" throughout the manuscript, as L. 54, 55, 78, 140,389, 455, 457, 587, 595, 648.
We thank the reviewer for this suggestion. The terminology has been revised throughout the manuscript, and "lytic phages" and "strictly lytic phages" have been replaced with "virulent phages" where appropriate (e.g., lines 53, 54, 77, 137, 381, 443, 445, 573, 580, and 634 in the revised manuscript).

• L.58: I recommend to replace "compare to the smaller genome phages by "compare to the five smaller genome phages"
Corrected as suggested (line 57).

• L.111: I would reformulate "Indeed, phages present unique..." by "Indeed, phages, and specifically virulent phages, present unique..."
The sentence has been revised as suggested (lines 109-110).

• L.145: replace 3 by three
Corrected (line 143). Numerical values below 10 have been consistently written in words throughout the manuscript where appropriate (e.g., lines 226, 485).

• L.160 and 162: is the difference of CaCl2 concentration ok?
We thank the reviewer for this comment. The difference in $CaCl_2$ concentration reflects optimization of experimental conditions for the respective assays.

• L.225: replace blastn by Blastn
Corrected in both the main manuscript and the Supplementary Materials and Methods (lines 222 and 98, respectively).

• L.228: replace lytic host range by plaquing host range
Corrected in both the main manuscript and the Supplementary Materials and Methods (lines 225 and 100, respectively).

• L.308: replace structures by morphotypes
Corrected (line 303).

• L.339: I would write "...average genome depth coverage..."
Revised as suggested (line 333).

• L.363: replace 7 by seven. Same L.621
Corrected (line 356 and line 606).

• L.443 and 444: I recommend not using the terms "phylogenetic relationships" and "phylogenetic tree" but the terms whole proteomic relationships and proteomic dendrogram, given VIPTree is based on whole proteome similarity. Same comment concerning FigS4 legend.

We thank the reviewer for this important suggestion. The terminology has been revised throughout the manuscript (lines 432-433), including in the legend of the Figure S4 (lines 998-1006).

• L.485: I would write "... protein families..."
Revised as suggested (line 473).

• L.487: I recommend qualifying a bit the statement, by writing "..., potentially pointing..."
The statement has been revised to include "potentially" as suggested (line 475).

• L.556: I would write "jumbo myophages" instead of "myovirus jumbophages", as you do L.560. In this case, the word myoviruses (or myophages) does not need to be in italics.
The terminology has been revised to "jumbo myophages", and italics have been removed as suggested (lines 542 and 547).

• L.597: For is in bold, remove this.
Corrected (line 583).

• L.639: I would write "...eight novel virulent phages..." and "...the first three..."
Corrected as suggested (line 625).

• L.644: I would write "...the five smaller genome phages..."
Corrected as suggested (line 630).

Response to Reviewer #2

Reviewer #2 (Comments for the Author):

The manuscript describes eight novel lytic tailed phages isolated from wastewater samples, including three jumbophages, which are the first known to infect all X. hortorum pv. vitians. These jumbophages define a new viral genus and exhibit distinct biological characteristics, including broader host ranges, but lower burst sizes and slower lytic cycles compared to the smaller-genome phages.
The newly isolated phages exhibited lytic activity against nearly all X. hortorum pv.vitians strains tested and were highly specific to the pathovar. Genomic analyses confirmed lytic profiles, and absence of virulence or resistance genes, supporting their biosafety for agricultural use. The phages demonstrated high stability across a wide range of pH and temperature conditions, remaining highly sensitive to UV exposure. These findings provide a valuable foundation for developing phage-based biocontrol strategies against bacterial leaf spot of lettuce.

The manuscript has been improved according to Reviewers 1 and 2 comments. There is only one advice for this text:

Line 102 "Their potential was recognized as early as 1924, when Hemstreet and Mallmann used a filtrate from decomposed cabbage containing phages to treat Xanthomonas campestris pv. campestris (12).

It is a general mistake, met in several reviewed manuscripts: In reference 12 Mallmann W.L, Hemstreet C. 1924. Isolation of an inhibitory substance from plants., p. 599-602 698 pp.. Journal of Agricultural Research, the Authors have described bacteriophages of soft rot pathogens, not of the black rot agent Xanthomonas campestris.

We thank the reviewer for the positive evaluation of our manuscript and for the helpful comment.

Citation from Mallmann W.L, Hemstreet C. (1924): "Pure cultures were obtained of Bacillus coli, B. carotovorus, B. melonis, and an organism closely resembling B. carotovorus but apparently belonging to the fluorescent group, which is responsible for a soft rot of cabbage. Varying amounts (up to 2 c.c.) of the cabbage filtrate rot were added after passing through a Berkefeld filter to each of these cultures, and subsequently to those

of B. spieckermani and the 'potato rot bacillus'. The inhibitory effects of the filtrate on the cabbage rot organism were immediately apparent, growth being retarded for 48 hours. After repeated transfers the inhibitory substance also adapted itself to B. spieckermani and B. carotovorus but produced no effect on the potato rot organism.". Please, correct this sentence.

We thank the reviewer for pointing out this inaccuracy. The sentence has been corrected to reflect that Hemstreet and Mallmann (1924) described bacteriophage activity against soft rot-associated bacteria rather than *Xanthomonas campestris*. The revised sentence now reads: "Their potential was recognized as early as 1924, when Hemstreet and Mallmann used a filtrate from decomposed cabbage containing phages to inhibit soft rot-associated bacteria (12)." (lines 100-102).

Re: Spectrum00846-26R1 (Genomic and phenotypic characterization of novel jumbo and small bacteriophages infecting Xanthomonas hortorum pv. vitians: Toward a phage-based biocontrol strategy)

Dear Dr. Frank Bertolla:

Your manuscript has been accepted, and I am forwarding it to the ASM production staff for publication. Your paper will first be checked to make sure all elements meet the technical requirements. ASM staff will contact you if anything needs to be revised before copyediting and production can begin. Otherwise, you will be notified when your proofs are ready to be viewed.

Data Availability: ASM policy requires that data be available to the public upon online posting of the article, so please verify all links to sequence records, if present, and make sure that each number retrieves the full record of the data. If a new accession number is not linked or a link is broken, provide production staff with the correct URL for the record. If the accession numbers for new data are not publicly accessible before the expected online posting of the article, publication may be delayed; please contact ASM production staff immediately with the expected release date.

Publication Fees: For information on publication fees and which article types have charges, please visit our website. We have partnered with Copyright Clearance Center (CCC) to collect author charges. If fees apply to your paper, you will receive a message from no-reply@copyright.com with further instructions. For questions related to paying charges through RightsLink, please contact CCC at ASM_Support@copyright.com or toll free at +1-877-622-5543. CCC makes every attempt to respond to all emails within 24 hours.

ASM Membership: Corresponding authors may join or renew ASM membership to obtain discounts on publication fees. Need to upgrade your membership level? Please contact Customer Service at Service@asmusa.org.

Embargo Policy: A press release may be issued as soon as the manuscript is posted on the Spectrum Latest Articles webpage. The corresponding author will receive an email with the subject line "ASM Journals Author Services Notification" when the article is available online.

The ASM Journals program strives for constant improvement in our submission and publication process. Please tell us how we can improve your experience by taking this quick Author Survey.

Thank you for submitting your paper to Spectrum.

Sincerely,
Olaya Rendueles
Editor
Microbiology Spectrum
